# Dietary supplement of *Smilax china* L. ethanol extract alleviates the lipid accumulation by activating AMPK pathways in high-fat diet fed mice

**DOI:** 10.1186/s12986-019-0333-z

**Published:** 2019-01-21

**Authors:** Licong Yang, Yan Zhao, Yongfang Pan, Dongming Li, Guodong Zheng

**Affiliations:** Jiangxi Key Laboratory of Natural Product and Functional Food, College of Food Science and Engineering, Jiangxi Agricultural University, Nanchang, 330045 China

**Keywords:** *Smilax china* L. ethanol extract, Obesity, AMP-activated protein kinase, Lipid metabolism, Sterol regulatory element-binding proteins

## Abstract

**Background:**

Obesity has become a public health concern worldwide because it is linked to numerous metabolic disorders, such as hyperlipidemia, hypertension and cardiovascular disease. Therefore, there is an urgent need to develop new therapeutic strategies that are efficacious and have minimal side effects in obesity treatment. This study examined the effect of dietary supplement of *Smilax china* L. ethanol extract (SCLE) on high-fat diet (HFD) induced obesity.

**Methods:**

Fifty ICR mice were fed a normal diet, high-fat diet (HFD) or HFD supplemented with 0.25, 0.5% or 1% SCLE for 8 weeks. Body weight, intraperitioneal adipose tissue (IPAT) weight, serum biochemical parameters, and liver lipids were measured. Activity, mRNA and protein expressions of lipid metabolism-related enzymes were analyzed.

**Results:**

Over 0.5% SCLE had reduced cholesterol biosynthesis by the activation of AMP-activated protein kinase (AMPK), which subsequently suppressed the mRNA expression of both sterol regulatory element binding protein-2 and 3-hydroxy-3-methyl-glutaryl-CoA reductase. Thus, the plasma and liver cholesterol concentrations in the HFD-fed mice were decreased. AMPK activation caused by SCLE also significantly upregulated lipolysis by enhancing adipose triglyceride lipase and hormone-sensitive lipase activities. This accelerated triglyceride hydrolysis and fatty acid release. Finally, SCLE increased carnitine palmitoyltransferase 1 and acyl-CoA oxidase activities, which further promoted fatty acid β-oxidation.

**Conclusion:**

SCLE could lead to a decrease in body weight gain and fat mass by inhibiting the lipid synthesis and promoting lipolysis and β-oxidation in HFD fed mice. The underlying mechanism is probably associated with regulating AMPK pathway.

**Electronic supplementary material:**

The online version of this article (10.1186/s12986-019-0333-z) contains supplementary material, which is available to authorized users.

## Introduction

With increasing westernization of food habits and lifestyle, obesity in infancy and adolescence has become a public health concern worldwide [1]. There is compelling evidence that suggests obesity is linked to the development of numerous metabolic disorders, such as diabetes with insulin resistance, hyperlipidemia, hypertension, cardiovascular disease, osteoarthritis and some cancers [2–4]. Lifestyle changes in dietary intake and physical activity contribute to the development of obesity [5]. The primary recommendations to the treatment of obesity include increasing physical activity and reducing calorie intake [6]. Anti-obesity drug is only necessary when the behavioral approach is not sufficient to get the optimal target of weight and metabolic control. Moreover, these drugs can lead to short-term weight loss, and patients have difficulty in maintaining a normal weight over time due to lack of compliance to their drug regimen [7].

Given these problems with obesity treatment, the popularity of dietary supplements for use in weight management has increased. Dietary supplements, which include vitamins, minerals, herbs, and amino acids, are widely used by adults and children of all ages. In particular, herbal supplements are commonly used around the world, either in place of or to supplement conventional (Western) medical therapies. Recent food science research has focused on identifying active plant ingredients that can suppress lipid accumulation with no side effects. Moreover, it has been reported that AMP-activated protein kinase (AMPK) can be regulated by natural plant compounds such as berberine, resveratrol, catechin epigallocatechin-3-gallate and puerarin [8–10].

AMPK and sterol regulatory element binding proteins (SREBPs) are known to participate in adipogenesis. AMPK is a serine/threonine protein kinase that functions as a metabolic master switch [11]. It is expressed in a number of mammalian organs, such as liver and adipose tissue. AMPK activation depends on phosphorylation of Thr172 on its α subunit [12]. AMPK is activated when ATP consumption causes an increase in the AMP:ATP ratio [13]. This activation alters cellular metabolism by activating ATP generating pathways, while blocking ATP consuming pathways [14]. Due to this, once activated, it regulates a large number of metabolic processes, including stimulation of ATP generation such as fatty acid oxidation, inhibition of ATP consumption such as fatty acid, cholesterol, and protein synthesis [15]. The acute actions of AMPK are partly due to direct regulation of the activity of target proteins, such as various metabolic enzymes including fatty acid synthase (FAS), adipose triglyceride lipase (ATGL), and hormone-sensitive lipase (HSL) [14, 16, 17]. Moreover, it can phosphorylate critical transcription factors that regulate the expression of metabolically-responsive target genes. It has been reported that in long-term regulation, AMPK can regulate hepatic lipogenic gene expression by inhibiting transcription factors such as SREBPs. This ultimately leads to a reduction in the overall rate of transcription [18]. SREBPs are well-known nuclear transcription factors that are involved in the biosynthesis of cholesterol, triglycerides and fatty acids in mammals [19]. This suggests that inhibition of the SREBP pathway might decrease fat accumulation [20]. Based on its underlying mechanism and outward effects, AMPK has often been reported as a potential therapeutic target for the treatment of obesity. Given this, herbal supplements that target and regulate metabolic enzyme gene expression could provide a means for significant, long-term weight loss in obese patients.

*Smilax china* L. is a climbing plant species and a well-known Chinese traditional medicine in the genus *Smilax*. It grows in the southern parts of China, Korea, Japan, Philippines, Vietnam, Thailand, Myanmar, and Assam. Previous research has shown that *Smilax china* L. derived compound extract has various pharmacological properties, including anti-inflammatory [21], anti-cancer [22] and anti-oxidant properties [23]. The main active components of *Smilax china* L. are stilbenes, flavonoids, polyphenols and steroidal saponins [24, 25]. Polyphenol compounds like resveratrol are also found in *Smilax china* L. Critically they have been found to have anti-obesity effects including the inhibition of adipocyte differentiation and fat accumulation [26]. Recently, an in vitro study showed that the water-soluble fraction of ethanol extracted *Smilax china* L. leaf decreased lipid accumulation in 3 T3-L1 adipocytes [27]. However, the in vivo anti-obesity effects and mechanisms of *Smilax china* L. are still un-known. Therefore, in this study, we sought to investigate the effect of *Smilax china* L. ethanol extract (SCLE) on body weight, intraperitoneal adipose tissue (IPAT), serum biochemical parameters, activated AMPK in liver, and regulation of SREBP-2 expression in high-fat diet (HFD)-fed mice. We also explored its effects on the mRNA and protein levels of hepatic and IPAT lipid metabolic enzymes in HFD-fed mice.

## Materials and methods

### Extraction and composition analysis

The *Smilax china* L. root was collected from Anhui province, China, provided by Simcere Drugstore in Nanjing, China. The dry powder of *Smilax china* L. was soaked in 60% EtOH (1:20) for 2 h. The solution was ultrasonically extracted for 30 min. After extraction, the supernatant was separated by filtration, followed by evaporation and lyophilization. The content of major components and polyphenols in SCLE was identified by UV spectroscopy and High Performance Liquid Chromatography (HPLC), respectively. The contents of total triterpenoid, total polyphenol and total flavonoids in the lyophilized ethanolic extract (SCLE) were 14.03 ± 0.21, 42.54 ± 0.45 and 29.69 ± 0.37%, respectively. The main components of polyphenol in SCLE were assessed by HPLC. Among the polyphenols, the main active components were chlorogenic acid, astilbin, engeletin and resveratrol. SCLE contained 13.65 ± 0.19, 23.65 ± 0.36, 7.97 ± 0.03 and 7.46 ± 0.15 mg/g of chlorogenic acid, astilbin, engeletin and resveratrol, respectively (Additional file 1: Tables S1, S2 and Figure S1).

### Animal and diets

Four-week-old female ICR mice, weighing about 20 g, were purchased from Hunan Silaike Laboratory Animal Co., Ltd. (Changsha, China). All mice were acclimated on a standard AIN-93 G diet for one week. Then fifty mice were weight-matched and distributed into five groups orally administered with normal diet, HFD and HFD plus 0.25, 0.5% or 1% SCLE for 8 weeks. The HFD contained 42.2% carbohydrates, 21% protein, and 27% fat. The percentage of calories from fat in the HFD is 49%, and the percentages of saturated, monounsaturated and polyunsaturated fat acid in total fat acid are 54.5, 29.6 and 15.9%, respectively. The composition of the diets was shown in Additional file 1: Table S3. Mice were housed in standard cage conditions at a constant temperature (24 ± 2 °C), humidity 50 ± 10% and a 12 h light-dark cycle (lights on from 08:00 to 20:00), with food and tap water available ad libitum. During the feeding period, mice were weighed every week and food intake was measured every day. At the end of feeding period, mice were fasted for 12 h before sacrifice. Blood was drawn from heart and clotted at the room temperature. Then, serum was isolated by centrifugation at 825 g for 15 min at 4 °C, and immediately stored at − 80 °C. The liver, spleen, kidney and IPAT (including the periuterine adipose tissue, perinephric adipose tissue and mesenteric adipose tissue) of each mouse were harvested and weighed. Then livers and IPAT were cut into five pieces, washed with saline, and frozen at − 80 °C.

This study was carried out in strict accordance with the recommendations from the Guide for the Care and Use of Laboratory Animals of the Chinese Association for Laboratory Animal Science. All animal care and protocols were approved by the Animal Care and Use Committee of the Jiangxi Agricultural University. All killings were performed under sodium pentobarbital anesthesia, and efforts were taken to minimize animal suffering.

### Biochemical analysis

Serum levels of triglycerides (TG), total cholesterol (TC), high-density lipoprotein cholesterol (HDL-C) and low-density lipoprotein cholesterol (LDL-C) were analyzed using commercial kits (Biosino Biotechnology and Science Inc., Beijing, China) according to the manufacturer’s instructions. The total lipids in the liver were extracted by the method of Folch et al [28]. TG and TC contents of the liver were also analyzed using commercial kits (Biosino Biotechnology and Science Inc., Beijing, China).

### Measurement of liver lipid metabolism related enzyme acticvities

The frozen liver was homogenized in buffer A (3 mmol/L Tris/HCl, pH 7.2, 1 mmol/L EDTA, 1 mmol/L dithiothreitol, 25 μmol/L ALLN (N-acetyl-leucyl-leucyl-norl-leucina, a calpain and cathepsin inhibitor), 100 μmol/L leupeptin, 100 μmol/L AEBSF (4-(2-aminoethy1) bezenesulfonyl-fluoride, a serine protease inhibitor), 10 μmol/L E64, and 0.25 mol/L sucrose) by the method of Moriyama et al [29]. Protein concentration of the homogenate was measured using a kit based on the Coomassie brilliant blue method (#A045–2; NanJing Jian Cheng Bioengineering Institute, NanJing, China) and then adjusted to 10 mg/mL for carnitine palmitoyltransferase 1 (CAT1) activity analysis. The homogenate was centrifuged at 500 g for 10 min and the supernatant was used in assays for acyl-CoA oxidase (ACO) activity. The supernatant (500 g) was further centrifuged at 9000 g for 15 min and this supernatant was used for the analysis of fatty acid synthase (FAS) and AMP-activated protein kinase (AMPK) activities. The activities of CAT1, ACO, FAS and AMPK in the liver were determined by the method of Zhao et al [10].

### Measurement of mRNA expression by real-time quantitative PCR

Total RNA from frozen liver and IPAT were prepared by using Trizol (Invitrogen) according to the manufacturer’s instructions. Single-strand cDNA synthesis was performed using the cDNA Reverse Transcription Kit (Takara Bio Inc., Japan) according to the manufacturer’s protocols. Real-time quantitative PCR was performed using an Applied Biosystems 7900HT Real-Time PCR System (Applied Biosystems, USA) and Premix Ex Taq (Probe qPCR). Briefly, PCR was performed in a final volume of 20 μL containing 800 ng of cDNA, 0.4 μL of 0.4 mmol/L forward and reverse primers, 0.4 μL of 0.4 mmol/L fluorescence probe, 10 μL of 1.25 U/25 μL Premix Ex Taq, and 0.4 μL of 25 μmol/L ROX Reference Dye. PCR reactions consisted of an initial denaturing step at 95 °C for 30s, followed by 45 cycles of 10 s at 94 °C and 37 s at 60 °C. The primers and probes used are given in Table 1. Results are presented as levels of expression relative to those of controls after normalization to glyceraldehyde 3-phosphate dehydrogenase (GADPH) using 2^-ΔΔCT^ method.

**Table 1:** The PCR Primer and Probe sequence of different genes

Gene	Forward primer	Reverse primer
FAS	5’-GGGCTCTATGGATTACC-3′	5’-CATAGCTGACTTCCAACA-3′
HMG-COAR	5’-GAATGCCTTGTGATTGGAGTTG-3′	5’-GCCGAAGCAGCACATGATCT-3′
HSL	5’-GGAGCACTACAAACGCAACGA-3′	5’-TCGGCCACCGGTAAAGAG-3′
ATGL	5′- CGCGCTCTTGGCTCATG − 3′	5′- CCAACCTTTGTGCCCCTTAA-3′
CAT	5’-CTGTGGGATGGTGTATGAGCAT-3′	5’-GACATGGTCCACAAGTGCAACT-3′
ACO	5′- TCACAGCAGTGGGATTCCAA − 3’	5’-TCTGCAGCATCATAACAGTGTTCTC-3’
AMPK	5’-TGAAGATCGGCC ACTACATCCT-3’	5’-CTTGCCCACCTTCACTTTCC-3’
SREBP-2	5’-CCCTTCAGTGCAACGGTCATTCAC-3’	5′- TGCCATTGGCCGTTTGTGTC-3’
GADPH	5’-TGTGTCCGTCGTGGATCTGA-3’	5’-CCTGCTTCACCACCTTCTTGA-3’

### Measurement of the protein expression

The frozen liver and IPAT were homogenized separately in liquid nitrogen and lysed in a radio immunoprecipitation assay buffer (50 mM Tris, pH 7.4, 150 mM NaCl, 1% Triton X-100, 0.5% sodium deoxycholate, 0.1% SDS, 1 mM-EDTA, 1 mM-phenylmethanesulphonyl fluoride and 2 mg/mL leupeptin) at 4 °C for 1 h. Then the liver and IPAT lysates were centrifuged at 9000 g for 15 min and the supernatant was used for furthermore experiment. Equal amounts of proteins were heated in SDS loading buffer at 100 °C for 10 min, separated on a 8% SDS-polyacrylamide gel and transferred onto a polyvinylidene difluoride (PVDF) membrane (Millipore, MA, USA). The membrane was blocked with 5% non-fat milk in PBST (137 mM NaCl, 2.7 mM KCl, 10 mM Na_2_HPO_4_, 2 mM KH_2_PO_4_, 0.1% Tween-20, pH 7.4) at room temperature for 2 h. After block, the membranes were incubated at 4 °C overnight with 1:1000 dilutions of primary antibodies against AMPKα (#2793; Cell Signaling Technology Inc. MA, USA), phosphorylated AMPKα (p-AMPKα) (Thr172) (#2535, Cell Signaling Technology Inc. MA, USA), FAS (#3180; Cell Signaling Technology Inc. MA, USA), ATGL (#ab85858; Abcam Limited, Cambridge, UK), HSL (#4107; Cell Signaling Technology Inc. MA, USA) and phosphorylation of HSL Ser660 (p-HSL (Ser660)) (#4126, Cell Signaling Technology Inc. MA, USA). Then the membranes were washed three times with PBST and then incubated with secondary horseradish peroxidase-conjugated antibodies (ZSGB-Bio., Beijing, China) at room temperature for 1 h. The transferred proteins were visualized with an enhanced 3, 3′-diaminobenzidine tetrahydrochloride kit (ZSGB-Bio., Beijing, China). Then, the “Image-Pro Plus” software was used to analyze the OD of protein band.

### Statistical analysis

The data obtained in this study were analyzed using SPSS 17.0 software (SPSS Inc., Chicago, USA). All values are expressed as means ± standard error of the mean (SEM). Continuous variables were tested for normality, and intergroup differences were analyzed by one-way ANOVA, followed by usage of Tukey’s post hoc test to compare the group means. A *P* value < 0.05 was viewed significant.

## Results

### SCLE ameliorates metabolic abnormalities in high-fat diet induced obese mice

**Fig. 1:**
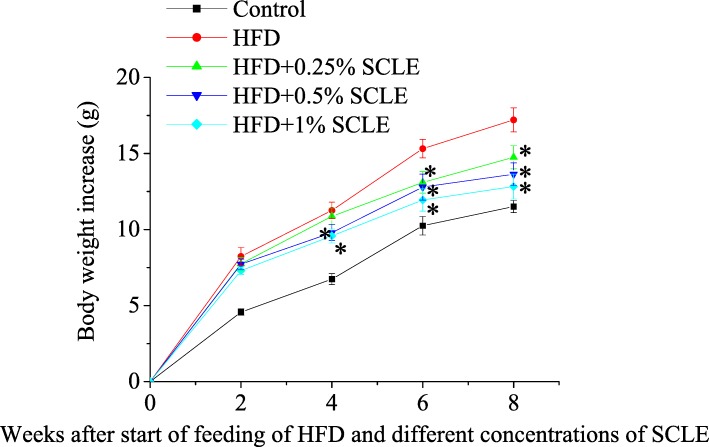
Effect of SCLE on the body weight gain in mice (g). The body weight gain of the HFD was significantly higher than that of the control from second week to the end of this experiment. In contrast, 0.25% SCLE significantly decreased the body weight gain from week 6. Also, 0.5 and 1.0% SCLE significantly decreased the body weight gain of high-fat diet induced obese mice at the fourth week. Values are means ± SEM of 10 mice. *: Significant difference at *p* < 0.05 compared to the HFD. The body weight gain of the HFD group was significantly higher than that of the control from second week to the end of this experiment. At the sixth week, the body weight gain of 1.0% SCLE-treated group was normally to control. These results are not shown in Fig. 1

The body weight gain of the HFD was significantly higher than that of the control from second week to the end of the experimental timeline (Fig. 1). In contrast, the body weight gains were significantly decreased by 0.25% SCLE from Week 6, whereas at Week 4 by 0.5 and 1.0% SCLE treatment. During the treatment period, energy intake in HFD was similar with the HFD supplement with SCLE (Table 2). It is notable that HFD-fed mice receiving diets supplemented with over 0.5% SCLE had significantly lower IPAT weight. However, there were no significant differences in liver, spleen or kidney weights between all groups.

**Table 2:** Effect of SCLE on energy intake, weight of organs and IPAT in mice

	Energy intake (kcal/day)	Liver (mg)	Spleen (mg)	Kidney (mg)	IPAT(mg)
Control	20.49 ± 1.27^b^	1543 ± 48.9^a^	135.6 ± 6.4^a^	406.0 ± 20.1^a^	1322 ± 52.1^c^
HFD	26.47 ± 1.89^a^	1522 ± 44.3^a^	146.7 ± 5.4^a^	395.5 ± 13.8^a^	2133 ± 93.9^a^
HFD + 0.25% SCLE	26.17 ± 1.97^a^	1525 ± 47.0^a^	130.9 ± 9.4^a^	389.1 ± 10.8^a^	1817 ± 98.1^ab^
HFD + 0.5% SCLE	25.84 ± 1.84^a^	1682 ± 80.8^a^	133.8 ± 8.6^a^	413.1 ± 23.1^a^	1675 ± 57.5^b^
HFD + 1.0% SCLE	25.69 ± 1.67^a^	1623 ± 62.7^a^	142.6 ± 8.5^a^	384.1 ± 14.4^a^	1561 ± 50.8^bc^

Values are means ± SEM of 10 mice. Means within the same column but not sharing the same superscript letter are significantly different (*P* < 0.05)

Additional file 1: Table S3. Composition of the diets (g/kg)

Serum and liver lipid levels were then analyzed. As shown in Table 3, compared with HFD group, over 0.5% SCLE significantly decreased serum TC, TG and LDL-C levels. Meanwhile, hepatic TC and TG levels were markedly decreased by the same concentration SCLE treatment. The oil-O red staining of hepatic sections also revealed the lipid accumulation in HFD, and SCLE treatment significantly down-regulated the lipid in hepatic (Additional file 1: Figure S2). Taken together, these results show that SCLE can reduce lipid accumulation without decreasing energy intake in HFD fed obese mice.

**Table 3:** Effect of SCLE on the serum biochemical parameters and liver lipid levels in mice

	Control	HFD	HFD + 0.25% SCLE	HFD + 0.5% SCLE	HFD + 1.0% SCLE
Serum biochemical parameters (mmol/L)					
TC	1.87 ± 0.09^c^	3.03 ± 0.21^a^	2.57 ± 0.06^ab^	2.49 ± 0.16^b^	2.45 ± 0.13^b^
TG	1.49 ± 0.11^b^	2.53 ± 0.18^a^	1.40 ± 0.12^b^	1.42 ± 0.11^b^	1.57 ± 0.09^b^
HDL-C	1.10 ± 0.07^b^	1.43 ± 0.10^b^	2.15 ± 0.16^a^	2.45 ± 0.11^a^	2.22 ± 0.20^a^
LDL-C	0.79 ± 0.05^c^	1.53 ± 0.08^a^	1.26 ± 0.05^b^	1.20 ± 0.14^b^	1.19 ± 0.08^b^
Liver lipids (mmol/g liver)					
TC	7.37 ± 0.76^b^	13.82 ± 0.82^a^	10.18 ± 0.51^b^	9.23 ± 0.93^b^	7.93 ± 0.71^b^
TG	22.30 ± 2.55^b^	33.72 ± 1.37^a^	28.88 ± 2.96^ab^	26.79 ± 1.50^b^	25.44 ± 1.64^b^

Values are means ± SEM of 10 mice. Means within the same row but not sharing the same superscript letter are significantly different (*P* < 0.05)

*HDL-C*: high-density lipoprotein cholesterol; *LDL-C*: low-density lipoprotein cholesterol; *TC*: total cholesterol; *TG*, triglyceride

### SCLE regulates the activities of hepatic lipid metabolism-related enzymes

The activities of FAS, CAT1, ACO and AMPK in liver are shown in Table 4. Compared with HFD, the activities of CAT1 and ACO in mice fed 1.0% SCLE were significantly increased. AMPK activity was also markedly increased by supplementation with over 0.5% SCLE. However, the activity of FAS in HFD plus SCLE were identical to that of the HFD.

**Table 4:** Effect of SCLE on liver lipid metabolism related enzymes activity in liver (IU/mg protein)

	FAS	CAT	ACO	AMPK
Control	0.20 ± 0.01^a^	0.87 ± 0.05^a^	0.62 ± 0.02^c^	0.28 ± 0.01^ab^
HFD	0.19 ± 0.01^a^	0.70 ± 0.04^b^	1.02 ± 0.03^b^	0.25 ± 0.01^b^
HFD + 0.25% SCLE	0.19 ± 0.01^a^	0.72 ± 0.02^ab^	0.96 ± 0.07^b^	0.30 ± 0.01^ab^
HFD + 0.5% SCLE	0.16 ± 0.01^a^	0.82 ± 0.06^ab^	1.07 ± 0.05^b^	0.32 ± 0.03^a^
HFD + 1.0% SCLE	0.18 ± 0.01^a^	0.89 ± 0.05^a^	1.43 ± 0.02^a^	0.33 ± 0.02^a^

Values are means ± SEM of 10 mice. Means within the same column but not sharing the same superscript letter are significantly different (*P* < 0.05)

*ACC*: acetyl-CoA carboxylase; *FAS*: fatty acid synthase; *CAT*: carnitine acyltransferase; *ACO*: acyl-CoA oxidase; *AMPK*: AMP-activated protein kinase

### SCLE regulates the expression of metabolic genes

**Fig. 2:**
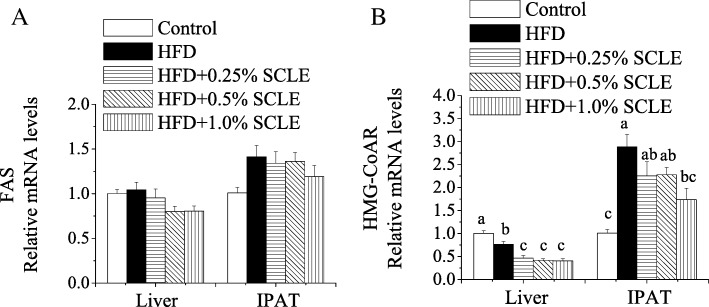
Effect of SCLE on lipogenic genes expression in liver and IPAT of mice. ICR mice were fed a HFD and SCLE for 8 weeks and their liver and IPAT tissues were obtained. The mRNA expression levels of FAS (**a**) and HMG-CoAR (**b**) related to lipogenesis were determined and compared between groups. Values are means ± SEM of 10 mice. Means within the same column but not sharing the same superscript letter are significantly different (*P* < 0.05)

The beneficial effect of SCLE on lipid metabolism was reflected in its effect on metabolic genes expression in hepatic and IPAT of mice. To determine whether SCLE could down-regulate the lipid synthesis and cholesterol biosynthesis, we examined FAS and HMG-CoAR mRNA levels, which were involved in lipogenesis. As shown in Fig. 2, 1.0% SCLE-treatment significantly decreased HMG-CoAR mRNA expression levels in hepatic and IPAT when compared with HFD. However, SCLE had no significant effect on either FAS mRNA levels.

**Fig. 3:**
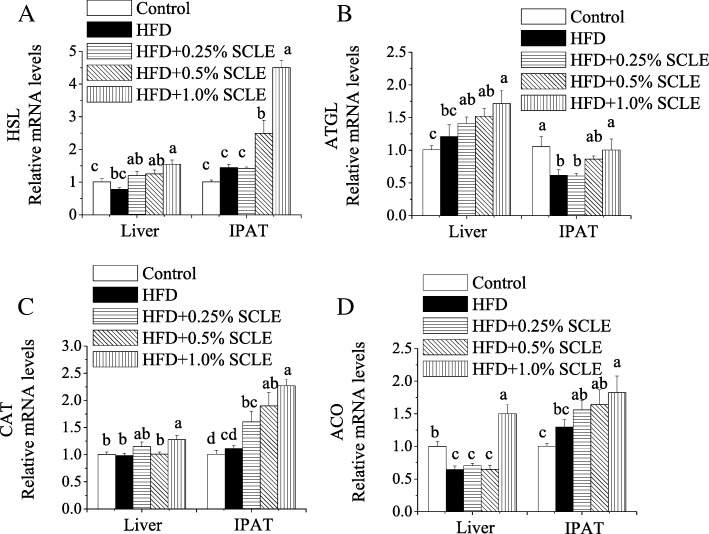
Effect of SCLE on lipolytic genes expression in liver and IPAT of mice. ICR mice were fed a HFD and SCLE for 8 weeks and their liver and IPAT tissues were obtained. The mRNA expression levels of HSL (**a**), ATGL (**b**), CAT (**c**) and ACO (**d**) related to lipolysis were determined and compared between groups. Values are means ± SEM of 10 mice. Means within the same column but not sharing the same superscript letter are significantly different (P < 0.05)

To investigate whether SCLE could accelerate lipolysis and fatty acid β-oxidation, we then examined HSL, ATGL, CAT1 and ACO mRNA levels. As shown in Fig. 3, compared to HFD, 1.0% SCLE significantly up-regulated HSL, ATGL, CAT1, and ACO mRNA expression levels in hepatic and IPAT.

**Fig. 4:**
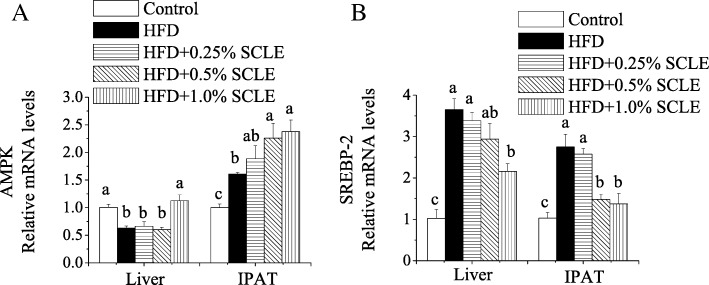
Effects of SCLE on mRNA expression of AMPK and SREBP-2 in the liver and IPAT of ICR mice. ICR mice were fed a HFD and SCLE for 8 weeks and their liver and IPAT tissues were obtained. The mRNA expression levels of AMPK (**a**) and SREBP-2 (**b**) were determined and compared between groups. Values are means ± SEM of 10 mice. Means within the same column but not sharing the same superscript letter are significantly different (P < 0.05)

mRNA levels of AMPK and sterol regulatory element binding protein-2 (SREBP-2) were also analyzed. As shown in Fig. 4, compared with the HFD, AMPK mRNA levels were dramatically increased by 1.0% SCLE in liver and IPAT, respectively. Meanwhile, SREBP-2 mRNA levels were significantly decreased by 1.0% SCLE in liver and IPAT, respectively.

### SCLE regulates proteins expression of metabolism-related genes in mice

**Fig. 5:**
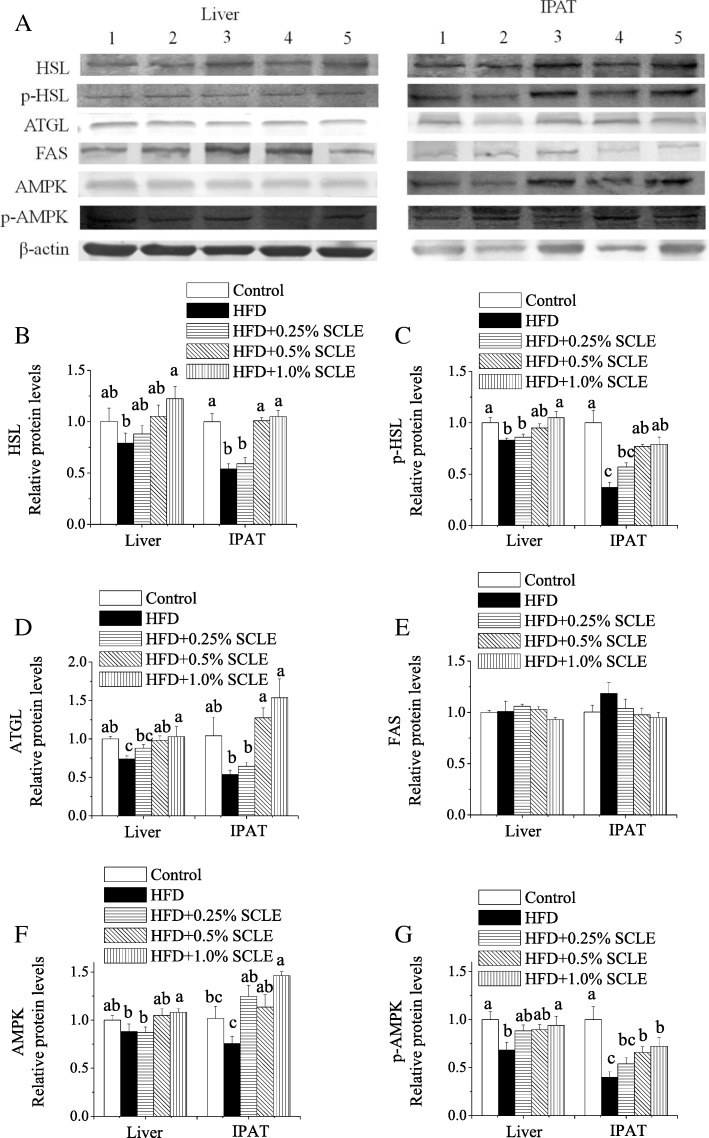
Effects of SCLE on the protein expression levels of lipid metabolism-related enzymes. Liver and IPAT were isolated from HFD and SCLE-fed ICR mice. **a** Western blot analysis of HSL, p-HSL, ATGL, FAS, AMPK, and p-AMPK in the liver and IPAT. Expression of HSL (**b**), p-HSL (**c**), ATGL (**d**), FAS (**e**), AMPK (**f**), and p-AMPK (**g**) normalized to β-actin and expressed relatively to control. Values are means ± SEM of 10 mice. Means within the same figure but not sharing the same superscript letter are significantly different (P < 0.05). 1, Control; 2, HFD; 3, HFD + 0.25% SCLE; 4, HFD + 0.5% SCLE; 5, HFD + 1.0% SCLE

Western blot results revealed that hepatic HSL, p-HSL, AMPK, and p-AMPK protein levels were significantly increased in animals receiving 1.0% SCLE when compared with HFD (Fig. 5). Furthermore, all levels were increased in IPAT of over 0.5% SCLE treated mice. 0.5 and 1.0% SCLE also markedly increased hepatic ATGL protein levels as well as the ATGL levels in IPAT. However, SCLE treatment had no effect on FAS levels in liver and IPAT, consistent with the mRNA expression levels.

## Discussion

The aim of the present study was to investigate the in vivo anti-obesity effects of SCLE dietary supplements. The results showed that SCLE reduced serum and hepatic lipid levels and thus led to the suppression of body weight gain and fat accumulation in the HFD-treated mice. Elevated TG and LDL-C levels are associated with an increased risk of atherogenic plaques formation. In our study, compared to HFD, serum TC, TG and LDL-C levels were markedly decreased by SCLE, indicating that SCLE can improve HFD-induced dyslipidemia. Moreover, to investigate the effects of SCLE on lipid metabolism, the activities of lipid metabolism-related enzymes were analyzed. The results showed that SCLE enhanced both the activity and expression of AMPK, CAT1, ACO, ATGL and HSL. In contrast, it reduced SREBP-2 and HMG-CoAR mRNA levels. HMG-CoAR is a key enzyme in cholesterol biosynthesis [30]. The activity of ATGL and HSL has been reported to be positively correlated with TG hydrolase activity in mouse white adipose tissue [31]. The enhancement of ACO and CAT1 activity (fat acid β-oxidation related enzymes) may contribute to the suppression of liver and visceral fat accumulation [32]. The results of the present study indicate that the enhancement of the activities of hydrolase (ATGL and HSL) and β-oxidation related enzymes (ACO and CAT1) may be the major reasons for the reduction of body weight gain in mice.

AMPK is a cellular sensor of energy metabolism and a regulator for cholesterol and hepatic lipid metabolism [33]. Activated AMPK can regulate hepatic lipogenic gene expression by inhibiting transcription factors such as SREBPs, thus reducing the overall transcription rate [30, 34]. There are two separate mammalian SREBP genes, SREBP-1 and -2. SREBP-1 targets genes involved in fatty acid metabolism, such as FAS [35]. SREBP-2 is responsible for cholesterol-related genes, e.g., HMG-CoAR (a key enzyme in cholesterol biosynthesis) [36, 37]. A very recent study also demonstrated that 5-aminoimidazole-4-carboxyamide ribonucleoside (AICAR)-induced activation of AMPK can directly inhibit the expression of SREBP-2 and its target genes HMG-CoAR and 3-hydroxy-3-methylglutaryl-Coenzyme A synthase (HMG-CoAS), which are key enzymes in cholesterol biosynthesis [30]. In the present study, SCLE significantly increased AMPK activity and decreased the SREBP-2 and HMG-CoAR expression levels. These results suggest that SCLE can enhance AMPK activity, thereby inhibiting cholesterol synthesis by suppressing SREBP-2 expression, resulting in low HMG-CoAR expression, which led to marked decreases in both serum and hepatic levels of TC and TG in SCLE treated mice.

Activated AMPK can also directly regulate the activity of target proteins, including various metabolic enzymes like ATGL and HSL [17, 38]. ATGL initiates triglyceride hydrolysis to yield diglyceride [39]. Diglyceride is then hydrolyzed by HSL to monoglyceride and subsequently, glycerol. At each stage, a fatty acid is released [40]. ATGL and HSL are two major mammal lipolytic enzymes, which account for more than 90% of TG hydrolase activity in mouse white adipose tissue [31]. Both enzymes are regulated by cAMP-mediated phosphorylation of perilipin [41]. Gaidhu et al. reported that AICAR induces AMPK activation, which promotes energy dissipation through induction of ATGL [17]. AMPK activation can also increase phosphorylation of HSL on Ser660, which results in HSL translocation to the lipid droplet [42]. In the present study, we found that SCLE can significantly enhance ATGL and HSL mRNA expression levels as well as markedly increase ATGL, HSL, and p-HSL protein expression, compared to the HFD. Taken together, these results suggest that SCLE-stimulated AMPK activation, potentially leads to HSL phosphorylation and up-regulated ATGL and HSL expression, which subsequently leads to triglyceride hydrolysis to fatty acids, resulting in decreased intracellular lipid droplet accumulation.

Liver is known as an organ active in β-oxidation. As such, up-regulation of hepatic lipid metabolism may contribute to the suppression of liver and visceral fat accumulation [32]. ACO and CAT1 are two important enzymes responsible for hepatic fatty acid β-oxidation. ACO catalyzes both the initial and rate-determining reactions in the β-oxidation of fatty acids. CAT1 catalyzes the reversible transfer of a fatty acyl-group from acyl-CoA to L-carnitine to facilitate the transport of acyl-moieties across plasma membranes. Previous research from our group has shown that long-term consumption of catechins and caffeine significantly enhanced CAT, ACO activities and mRNA expression as well as hepatic β-oxidation activity [10]. In our present study, we observed that SCLE increased the activities and mRNA expression of ACO and CAT1. These effects may enhance hepatic β-oxidation and lipolysis. Activated AMPK could then activate adenosine triphosphate-generating processes (e.g. fatty acid oxidation). Collectively, our results suggest that AMPK can promote the activity of CAT1.

## Conclusion

**Fig. 6:**
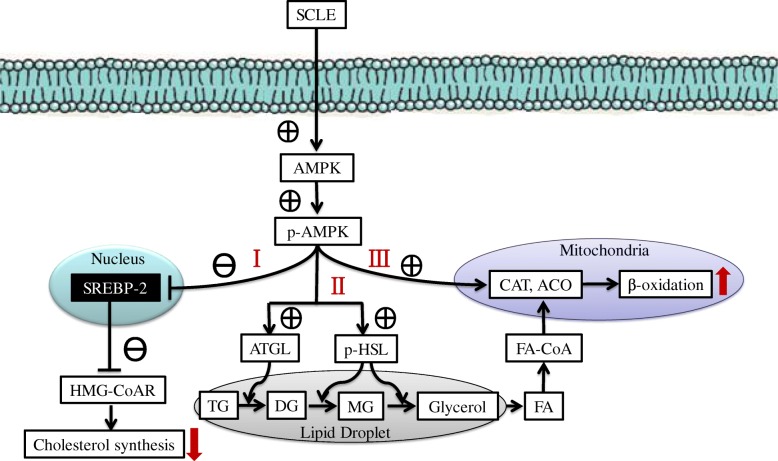
Proposed scheme illustrating the role of SCLE on lipid metabolism. SCLE possibly reduced lipid accumulation through the following process: I: increased AMPK phosphorylation to inhibit the expression of SREBP-2 and the target gene HMG-CoAR; II: the AMPK phosphorylation also stimulated the ATGL, HSL expression and the phosphorylation of HSL to modulate hydrolysis of triglyceride; III: enhanced the activity and expression of CAT and ACO to promote fatty acid β-oxidation. AMPK, AMP-activated protein kinase; p-AMPK; phosphorylated AMPK; SREBP-2, sterol regulatory element binding protein-2; HMG-CoAR, 3-hydroxy-3-methylglutaryl-Coenzyme A reductase; ATGL, adipose triglyceride lipase; p-HSL; phosphorylated HSL; CAT, carnitine acyltransferase; ACO, acyl-CoA oxidase; TG, triglyceride; DG, diglyceride; MG, monoglyceride; FA, fatty acid; FA-CoA, fatty acid CoA.  and → denote stimulation;  and ˨ denote inhibition

In summary, we provide evidence that SCLE protects against the development of obesity in HFD fed mice. A model for the effect of SCLE on lipid metabolism is proposed in Fig. 6. As demonstrated in our study, SCLE enhanced AMPK phosphorylation, which subsequently inhibited the expression of SREBP-2 and the target gene HMG-CoAR. As a result, cholesterol biosynthesis was decreased, resulting in lower serum and hepatic TC/TG levels than in HFD fed mice. Activated AMPK significantly up-regulated lipolysis by enhancing ATGL activity and HSL to p-HSL post-translational modifications. These changes in phosphorylation state then accelerated TG hydrolysis to release fatty acids. Moreover, p-AMPK increased CAT1 and ACO activities, further promoting fatty acid β-oxidation. This ultimately resulted in decreased body weight gain and IPAT weight. Taken together, SCLE can inhibit cholesterol synthesis and promote lipolysis and β-oxidation in HFD-fed mice. Furthermore, these effects may be related to activation of the AMPK signaling pathway. Although SCLE has been found to be effective at inhibiting body weight gain via AMPK activation, the exact component(s) in SCLE that made it effective are still unknown. Future work will be required to determine what component(s) of SCLE are responsible for its effectiveness. Due to the complexity of metabolism circumstance in human, more research on the application of SCLE in human is needed.

## Additional file

Additional file 1: **Table S1.** The content of major components in SCLE. Figure S1 Chromatograms of standard mixture (A) and SCLE (B). 1: Chlorogenic acid, 2: Astilbin, 3: Engeletin, 4: Resveratrol. Table S2 The content of active constituent in SCLE. Table S3 Composition of the diets (g/kg). Figure S2 Microscopic examination of oil-O red stained liver sections from mice (magnification 400×). (DOC 9302 kb)

## Abbreviations

ACO: Acyl-CoA oxidase
AMPK: AMP-activated protein kinase
ATGL: Adipose triglyceride lipase
CAT1: Carnitine palmitoyltransferase 1
FAS: Fatty acid synthase
HDL-C: High-density lipoprotein cholesterol
HFD: High-fat diet
HMG-CoAR: 3-hydroxy-3-methylglutaryl-CoA reductase
HMG-CoAS: 3-hydroxy-3-methylglutaryl-CoA synthase
HSL: Hormone-sensitive lipase
LDL-C: Low-density lipoprotein cholesterol
p-AMPK: Phosphorylated AMPK
p-HSL: Phosphorylated HSL
SCLE: *Smilax china* L. ethanol extract
SREBPs: Sterol regulatory element-binding proteins
TC: Total cholesterol
TG: Triglyceride

## References

[1] Rizzo AC, Goldberg TB, Silva CC, Kurokawa CS, Nunes HR, Corrente JE (2013). Metabolic syndrome risk factors in overweight, obese, and extremely obese brazilian adolescents. Nutr J.

[2] Nawrocki AR, Scherer PE (2005). The adipocyte as a drug discovery target. Drug Discov Today.

[3] Renehan AG, Tyson M, Egger M, Heller RF, Zwahlen M (2008). Body-mass index and incidence of Cancer: a systematic review and meta-analysis of prospective observational studies. Lancet.

[4] Zhang A, Sun H, Wang X (2013). Power of metabolomics in biomarker discovery and mining mechanisms of obesity. Obes Rev.

[5] Kremers S, Reubsaet A, Martens M, Gerards S, Jonkers R, Candel M, Weerdt ID (2009). Systematic prevention of overweight and obesity in adults: a qualitative and quantitative literature analysis. Obes Rev.

[6] Franz MJ, Bantle JP, Beebe CA, Brunzell JD, Chiasson JL, Garg A, Holzmeister LA, Hoogwerf B, Mayer-Davis E, Mooradian AD (2004). Nutrition principles and recommendations in diabetes. Diabetes Care.

[7] Derosa G, Maffioli P (2012). Anti-obesity drugs: a review about their effects and their safety. Expert Opin Drug Saf.

[8] Hardie D (2007). AMP-activated protein kinase as a drug target. Annu Rev Pharmaco.

[9] Zheng G, Lin L, Zhong S, Zhang Q, Li D (2015). Effects of Puerarin on lipid accumulation and metabolism in high-fat diet-fed mice. PLoS One.

[10] Zhao Y, Yang L, Huang Z, Lin L, Zheng G (2016). Synergistic effects of caffeine and catechins on lipid metabolism in chronically fed mice via the AMP-activated protein kinase signaling pathway. Eur J Nutr.

[11] Hardie DG, Ross FA, Hawley SA (2012). AMPK: a nutrient and energy sensor that maintains energy homeostasis. Nat Rev Mol Cell Bio.

[12] Hurley RL, Barré LK, Wood SD, Anderson KA, Kemp BE, Means AR, Witters LA (2006). Regulation of AMP-activated protein kinase by multisite phosphorylation in response to agents that elevate cellular cAMP. J Biol Chem.

[13] Xiao B, Heath R, Saiu P, Leiper FC, Leone P, Jing C, Walker PA, Haire L, Eccleston JF, Davis CT (2007). Structural basis for AMP binding to mammalian AMP-activated protein kinase. Nature.

[14] Mantovani J, Roy R (2011). Re-evaluating the general(ized) roles of AMPK in cellular metabolism. FEBS Lett.

[15] Hawley SA, Boudeau J, Reid JL, Mustard KJ, Udd L, Mäkelä TP, Alessi DR, Hardie DG, Hawley SA (2003). Complexes between the LKB1 tumor suppressor, STRAD/ and MO25/ are upstream kinases in the AMP-activated protein kinase cascade. J Biol.

[16] Winder WW, Holmes BF, Rubink DS, Jensen EB, Chen M, Holloszy JO (2000). Activation of AMP-activated protein kinase increases mitochondrial enzymes in skeletal muscle. J Appl Physiol.

[17] Gaidhu MP, Fediuc S, Anthony NM, So M, Mirpourian M, Perry RL, Ceddia RB (2009). Prolonged AICAR-induced AMP-kinase activation promotes energy dissipation in white adipocytes: novel mechanisms integrating HSL and ATGL. J Lipid Res.

[18] Viollet B, Foretz M, Guigas B, Horman S, Dentin R, Bertrand L, Hue L, Andreelli F (2006). Activation of AMP-activated protein kinase in the liver: a new strategy for the management of metabolic hepatic disorders. J Physiol.

[19] Goldstein JL, Debose-Boyd RA, Brown MS (2006). Protein sensors for membrane sterols. Cell.

[20] Xiao X, Song B-L (2013). SREBP: a novel therapeutic target. Acta Bioch Bioph Sin.

[21] Shu XS, Gao ZH, Yang XL (2006). Anti-inflammatory and anti-nociceptive activities of Smilax China L. aqueous extract. J Ethnopharmacol.

[22] Wu LS, Wang XJ, Wang H, Yang HW, Jia AQ, Ding Q (2010). Cytotoxic polyphenols against breast tumor cell in Smilax China L. J Ethnopharmacol.

[23] Kim KM, Suh JW, Yang SH, Kim BR, Park TS, Shim SM (2014). Smilax China root extract detoxifies nicotine by reducing reactive oxygen species and inducing CYP2A6. J Food Sci.

[24] Ruan HL, Zhang YH, Zhao W, Tan YF, Sun ZL, Wu JZ (2002). Studies on the chemical constituents of Smilax China L. Nat Prod Res Dev.

[25] Xu Y, Liang JY, Zou ZM (2008). Studies on chemical constituents of rhizomes of Smilax china. China journal of Chinese materia medica.

[26] Aguirre L, Fernández-Quintela A, Arias N, Portillo MP (2014). Resveratrol: anti-obesity mechanisms of action. Molecules.

[27] Yun HK, Kim KK, Kim DJ, Choe M (2015). Antiobesity effects of the water-soluble fraction of the ethanol extract of Smilax China L. leaf in 3T3-L1 adipocytes. Nutr Res Pract.

[28] Folch J, Lees M, Sloane Stanley GH (1957). A simple method for the isolation and purification of total lipids from animal tissue. J Biol Chem.

[29] Moriyama T, Kishimoto K, Nagai K, Urade R, Ogawa T, Utsumi S, Maruyama N, Maebuchi M (2004). Soybean beta-conglycinin diet suppresses serum triglyceride levels in normal and genetically obese mice by induction of beta-oxidation, downregulation of fatty acid synthase, and inhibition of triglyceride absorption. Biosci Biotechnol Biochem.

[30] Liu S, Jing F, Yu C, Gao L, Qin Y, Zhao J (2015). AICAR-induced activation of AMPK inhibits TSH/SREBP-2/HMGCR pathway in liver. PLoS One.

[31] Lord CC, Brown JM (2012). Distinct roles for alpha-beta hydrolase domain 5 (ABHD5/CGI-58) and adipose triglyceride lipase (ATGL/PNPLA2) in lipid metabolism and signaling. Adipocyte.

[32] Murase T, Nagasawa A, Suzuki J, Hase T, Tokimitsu I (2002). Beneficial effects of tea catechins on diet-induced obesity: stimulation of lipid catabolism in the liver. Int J Obesity.

[33] Carling D, Thornton C, Woods A, Sanders MJ (2012). AMP-activated protein kinase: new regulation, new roles?. Biochem J.

[34] Deng X, Dong Q, Bridges D, Raghow R, Park EA, Elam MB (2015). Docosahexaenoic acid inhibits proteolytic processing of sterol regulatory element-binding protein-1c (SREBP-1c) via activation of AMP-activated kinase. BBA-Mol Cell Biol L.

[35] Magaña MM, Osborne TF (1996). Two tandem binding sites for sterol regulatory element binding proteins are required for sterol regulation of fatty-acid synthase promoter. J Biol Chem.

[36] Osborne TF (1995). Transcriptional control mechanisms in the regulation of cholesterol balance. Crit Rev Eukar Gene.

[37] Goldstein JL, Brown MS (1990). Regulation of the mevalonate pathway. Nature.

[38] Chen WL, Chen YL, Chiang YM, Wang SG, Lee HM (2012). Fenofibrate lowers lipid accumulation in myotubes by modulating the PPARα/AMPK/FoxO1/ATGL pathway. Biochem Pharmacol.

[39] Zimmermann R, Strauss JG, Haemmerle G, Schoiswohl G, Birner-Gruenberger R, Riederer M, Lass A, Neuberger G, Eisenhaber F, Hermetter A (2004). Fat mobilization in adipose tissue is promoted by adipose triglyceride lipase. Science.

[40] Ammann M (2007). Regulation of lipolysis in adipocytes. Annu Rev Nutr.

[41] Miyoshi H, Nd PJ, Souza SC, Shen WJ, Zhang HH, Stancheva ZS, Kraemer FB, Obin MS, Greenberg AS (2007). Control of adipose triglyceride lipase action by serine 517 of perilipin a globally regulates protein kinase A-stimulated lipolysis in adipocytes. J Biol Chem.

[42] Holm C (2003). Molecular mechanisms regulating hormone-sensitive lipase and lipolysis. Biochem Soc T.

