# Supplementary material for: Dietary supplement of Smilax china L. ethanol extract alleviates the lipid accumulation by activating AMPK pathways in high-fat diet fed mice
Source: Nutr Metab (Lond). 2019 Jan 21;16:6. doi: 10.1186/s12986-019-0333-z (PMC6341655; doi:10.1186/s12986-019-0333-z)
Supplement: Supplementary file 1 — Table S1. The content of major components in SCLE. Figure S1 Chromatograms of standard mixture (A) and SCLE (B). 1: Chlorogenic acid, 2: Astilbin, 3: Engeletin, 4: Resveratrol. Table S2 The content of active constituent in SCLE. Table S3 Composition of the diets (g/kg). Figure S2 Microscopic examination of oil-O red stained liver sections from mice (magnification 400×). (DOC 9302 kb) [file 12986_2019_333_MOESM1_ESM.doc]

**Supplementary Information**

**Dietary Supplement of *Smilax china L.*** **Ethanol Extract Alleviates the Lipid Accumulation by Activating AMPK pathways in High-Fat Diet Fed Mice**

Licong Yang, Yan Zhao, Yongfang Pan, Dongming Li, Guodong Zheng*

Jiangxi Key Laboratory of Natural Product and Functional Food, College of Food Science and Engineering, Jiangxi Agricultural University, Nanchang, 330045, China

Corresponding to: Dr. Guodong ZHENG, Jiangxi Key Laboratory of Natural Product and Functional FoodJiangxi Key Laboratory of Natural Product and Functional Food, College of Food Science and Engineering, Jiangxi Agricultural University, Nanchang, 330045, China.

Tel & Fax: 86-791-83813863, E-mail: zrs150716@aliyun.com

Table S1. The content of major components in SCLE

| Ingredient | Total triterpenes | Total polyphenol | Total flavonoids |
| --- | --- | --- | --- |
| Content (%) | 14.03±0.21 | 42.54±0.45 | 29.69±0.37 |


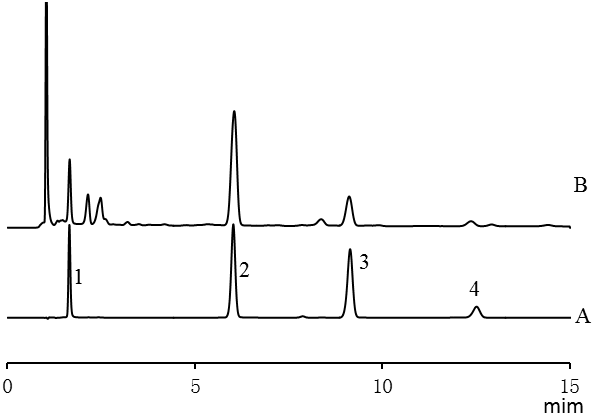


Figure S1. Chromatograms of standard mixture (A) and SCLE (B). 1: Chlorogenic acid, 2: Astilbin, 3: Engeletin, 4: Resveratrol

Table S2. The content of [active](javascript:void(0);) [constituent](javascript:void(0);) in SCLE

| Ingredient | Chlorogenic acid | | Astilbin | Engeletin | Resveratrol |
| --- | --- | --- | --- | --- | --- |
| Content (mg/g) | 13.65±0.19 | 23.65±0.36 | | 7.97±0.03 | 7.46±0.15 |

Table S3. Composition of the diets (g/kg)

| Ingredient | Control | HFD | HFD+0.25% SCLE | HFD+0.5% SCLE | HFD+1.0% SCLE |
| --- | --- | --- | --- | --- | --- |
| Corn starch | 398 | 195 | 192.5 | 190 | 185 |
| Dextrin | 127 | 127 | 127 | 127 | 127 |
| Sucrose | 100 | 100 | 100 | 100 | 100 |
| Cellulose | 50 | 50 | 50 | 50 | 50 |
| Casein | 210 | 210 | 210 | 210 | 210 |
| Vitamin mix.1 | 10 | 10 | 10 | 10 | 10 |
| Minaeral mix.2 | 30 | 30 | 30 | 30 | 30 |
| DL-methionine | 3 | 3 | 3 | 3 | 3 |
| Choline bitartrate | 2 | 2 | 2 | 2 | 2 |
| Corn oil | 70 | 70 | 70 | 70 | 70 |
| Beef tallow | — | 200 | 200 | 200 | 200 |
| Cholesterol | — | 3 | 3 | 3 | 3 |
| SCLE | — | — | 2.5 | 5 | 10 |
| Energy (kcal/kg) | 3970 | 4958 | 4948 | 4938 | 4918 |

^1,2^: Vitamin mixture and mineral mixture were prepared according to AIN-76TM.

Energy from sucrose, cornm starch, dextrin and casein were 4 kcal/g. Energy from corn oil and beef tallow were 9 kcal/g. Energy from cellulose, vitamin, mineral choline tartrate, cholesterol and SCLE was 0 kcal/g.

Figure S2. Microscopic examination of oil-O red stained liver sections from mice (magnification 400×).
